# Effect of database drift on network topology and enrichment analyses: a case study for RegulonDB

**DOI:** 10.1093/database/baw003

**Published:** 2016-03-15

**Authors:** Moritz E. Beber, Georgi Muskhelishvili, Marc-Thorsten Hütt

**Affiliations:** 1 Department of Life Sciences and Chemistry, Jacobs University, Campus Ring 1, Bremen 28759, Germany and; ^2^Bioinformatics Group, Max Planck Institute for Molecular Genetics, Ihnestraße 63–73, Berlin 14195, Germany

## Abstract

RegulonDB is a database storing the biological information behind the transcriptional regulatory network (TRN) of the bacterium *Escherichia coli*. It is one of the key bioinformatics resources for Systems Biology investigations of bacterial gene regulation. Like most biological databases, the content drifts with time, both due to the accumulation of new information and due to refinements in the underlying biological concepts. Conclusions based on previous database versions may no longer hold. Here, we study the change of some topological properties of the TRN of *E*. *coli*, as provided by RegulonDB across 16 versions, as well as a simple index, digital control strength, quantifying the match between gene expression profiles and the transcriptional regulatory networks. While many of network characteristics change dramatically across the different versions, the digital control strength remains rather robust and in tune with previous results for this index.

Our study shows that: (i) results derived from network topology should, when possible, be studied across a range of database versions, before detailed biological conclusions are derived, and (ii) resorting to simple indices, when interpreting high-throughput data from a network perspective, may help achieving a robustness of the findings against variation of the underlying biological information.

**Database URL:** www.regulondb.ccg.unam.mx

## Background

A typical task in bioinformatics is the evaluation of large data sets in the context of existing mechanistic knowledge and the functional interpretation of those data. The data sets are a close reflection of cell states and may be summarized as *omics* data. The principal access point to existing knowledge for bioinformatics research is a plethora of databases whose content range from primary sequence, via structural, to interaction information. The complex systems of cellular interactions are frequently represented as networks. Some common examples include protein–protein interaction networks, metabolic networks, transcriptional regulatory networks and various ontologies. A form of data interpretation is then a mapping of the experimental data onto a network representation of the database content. Enrichment analyses with respect to gene ontology (GO) classes or gene sets defined by signaling and metabolic pathways belong to this category (1).

There is a high level of awareness in the community about, on one hand, the problems of inter-experimental variation and thus reproducibility, and on the other hand, the lack of generalization of results from one experimental condition to another. Since knowledge is derived from experiments, databases suffer from many of the same problems. Equally important, our understanding of biology will remain incomplete for many years to come and databases can always ever deliver a momentary snapshot of our current understanding. In order to overcome the incomplete information inherent to any given database, many studies, e.g. (2, 3), relate their data either to multiple databases or consider a consensus representation of their content.

Integration of novel information and re-evaluation of existing information means that databases are prone to content drift. Hence, any results employing their content for data interpretation are just as transitory in nature—an implicit, yet often overlooked fact. In this study, we acknowledge the presence of this drift and, for the first time, investigate it systematically for a few examples of database-dependent data analyses.

At the heart of our investigation lies RegulonDB. The RegulonDB project has been a concerted effort to identify transcriptional regulatory interactions in *Escherichia coli* through literature review and, increasingly, specifically designed high-throughput experiments. There are several advantages that make RegulonDB ideal for this type of pilot study:

- RegulonDB has a rich, recorded history of almost two decades. It was first published in 1998 (4) and the latest major release was version 8.0 in 2013 (5). As of today, major and minor releases are available for download from version 4.0 until version 8.6 (4–12).
- Apart from a general increase in the number of identified transcription factors (TFs), TF binding sites and thus better promoter characterization, RegulonDB has had four major additions in content and must have undergone major reorganization in order to incorporate those additions: (i) operon organization (7–10), (ii) interactions with small ribonucleic acids (sRNA) (11), (iii) systematic information on σ-factors with releases 5.7 – 6.0 and (iv) annotation of gensor units (GUs), short for genetic sensory response units (12). A full description of changes is available on the web (http://regulondb.ccg.unam.mx/menu/about_regu londb/ whats_news/new_database_features.jsp), including simple summary statistics (http://regulondb.ccg.unam.mx/menu/about_regulondb/regulondb_history/da ta base_summary.jsp). A detailed explanation of the terms used and their meaning within the context of RegulonDB is available, too (http://regulondb.ccg.unam.mx/menu/using_regulondb/tutorials/project_glossary/index.jsp).
- The transcriptional regulatory network (TRN) of *E. coli* is one of the best investigated networks in systems biology. Hundreds of publications have been devoted to the analysis of its network architecture, see, e.g. (13–17). Additionally, the TRN has served as a frequent example illustrating methods from the statistical physics of complex networks, see, e.g. (18–21).
- Another, potentially more important, use of the TRN and of RegulonDB is the network-guided interpretation of gene expression data. This was pioneered by (22), where systematically different ‘activated’ topological structures were obtained for different cellular functions, such as cell cycle versus stress response. The investigation in (22) directly fits our previous description of a ‘typical bioinformatics task’.

Given the wealth of previous results compiled at different times within the last decade or so, we focus on revisiting a few exemplary ones. Our goal is to observe the outcomes of various methods with each RegulonDB release and assess their vulnerability or, conversely, stability in the presence of database drift.

Recent insights that cover the purely topological aspect of RegulonDB and similar biological networks are part of the general framework of ‘network biology’ (23): (i) Degree distributions of the TRN and the corresponding co-regulatory network (24) and their connection to the more general link between broad degree distributions of many biological networks and their potential impact on the robustness of the underlying biological systems (25). (ii) The over-representation (26) and functional role (27, 28) of feed-forward loops which again has found more general applications in the subgraph content (29, 30), where certain subgraphs are seen as devices encoding specific regulatory tasks (14). (iii) Hierarchy (23, 31) and its connection to essentiality (32). (iv) The importance of cycles and their orientation (33).

One of our own studies covers a second aspect of making use of RegulonDB: providing functional interpretation of gene expression data using network representations. In Ref (34), we introduced the concept of and a formalism for quantifying digital and analog control in bacterial gene regulation. The term ‘digital control’, on one hand, stands for the imprint which the TRN leaves in a given gene expression profile. The term ‘analog control’, on the other hand, denotes the imprint left by genome structure, i.e. the three dimensional organization of the circular bacterial chromosome, in the gene expression profile. In this way, we have discovered a tight interplay between digital and analog control governing gene expression patterns for a diverse set of perturbations of the gene regulatory machineries. The distinction of and coupling between digital and analog control has also been supported by a statistical analysis of gene locations (35).

## Results and discussion

There are four different network representations of biological relationships that are important to this study: (i) The TRN which consists of TFs and genes. It is thus a bipartite network that contains directed links from TFs to genes. These links encode four types of regulatory functions: activating, inhibiting, dual and unknown. The network may contain multiple links between the same two nodes with different interactions. (ii) Derived from the TRN, we also use the gene–gene regulatory network (GRN). This network is generated by connecting the genes that encode a specific TF with each target gene of that TF. We consider the GRN as a simple directed network, that means, the regulatory interaction information and multiple links are ignored. We do this to study the topology and the circular interactions that are only visible on this level. (iii) The TF intraconnections. The information on target genes from the TRN and which genes code for TFs are used to generate a network representation of how TFs regulate each other. This representation is used to investigate a hierarchy of TFs. (iv) The gene proximity network (GPN) is completely different. It is an undirected network where a link between two genes exists if they are located in close proximity to each other on the circular chromosome.

**Figure 1:**
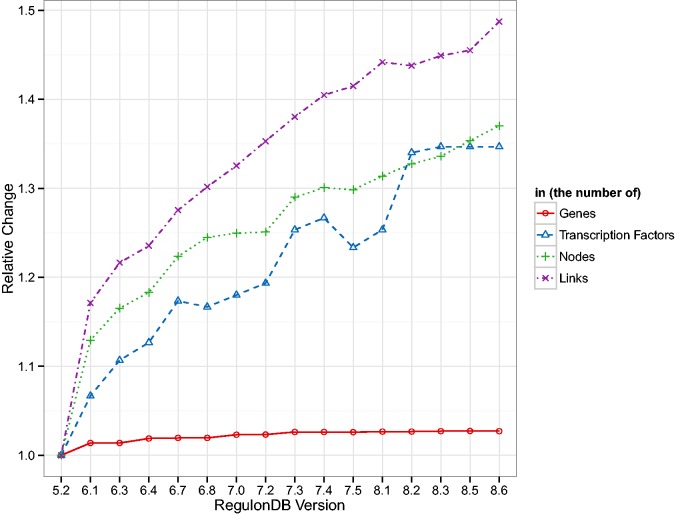
Shown are the number of genes and TFs present in RegulonDB and the number of nodes and links in the TRN normalized by the values in version 5.2. The number of genes is the most stable measure, whereas all other quantities show a clear upward trend. Interestingly, the number of nodes and links in the TRN increase in a similar fashion which leads to a general decrease in density of the TRN (cf. Supplementary Table A.1).

In order to assess how database drift can affect results and thus assertions about biological function, we tracked the basic change in topology of the TRN, GRN and GPN. In Figure 1 we show, normalized to their value in version 5.2, the general number of genes, TFs and nodes and links in the TRN. As can be expected, the number of genes remains quite stable throughout all database versions. Methods for identifying open reading frames (ORFs) are well established and their sequence and thus location on the chromosome can be assigned long before a function can to the respective transcripts. Thus it is unsurprising that the number of TFs increases as more and more DNA interactions are identified. The more regulatory interactions are verified, the more links, as well as potentially more TFs and genes, are included in the RegulonDB TRN. The added regulatory information are quite sparse, as can be seen in the decreasing density of the network (cf. Supplementary Table A.1). Since the genes and their locations change so little, we chose not to show any results related to the GPN but the results are present in Supplementary Table A.2 and Figures A.4, A.6 and A.7.

**Figure 2:**
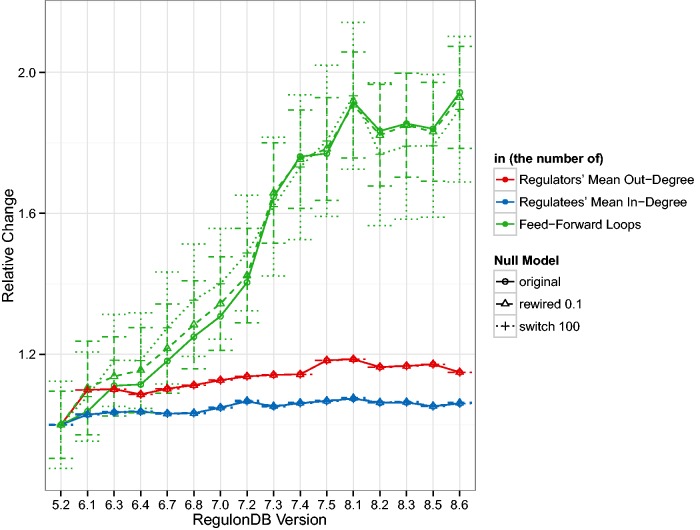
The curves represent the mean out-degree $\langle k_{\text{out}}\rangle$ of regulating nodes, i.e. those with $k_{\text{out}}>0$, the mean in-degree $\langle k_{\text{in}}\rangle$ of regulated nodes, that is, those with $k_{\text{out}}=0$, and the number of three-node feed-forward loops in the GRN normalized by the values for version 5.2. As reference values, we also show the mean and standard deviation of 1000 examples where 10% of the GRN links were rewired and 1000 examples where the GRN was switch-randomized. As expected the mean degree does not vary much, or at all, in the random networks but it is surprising that the mean relative number of feed-forward loops is so close to the original. The standard deviation denotes a fairly constant range of approximately 0.15–0.2 relative units. Despite a marked increase in the number of TFs over the years, and consequently an increase in both, the number of links and the number of source (TF) and target (gene) nodes (see Figure 1), $\langle k_{\text{in}}\rangle$ remains fairly stable over the years. There is about a 10% increase in the $\langle k_{\text{out}}\rangle$ which indicates that slightly more links have been added than new nodes introduced. Feed-forward loops have super-linear combinatorics, so their rapid increase due to an increasing number of links is not surprising but may indicate that more complicated dynamics may be present in the transcriptional regulatory system than previously considered. We were unable to identify any three-node feedback loops in any release, however.

Following the changes in the basic TRN topology, in Figure 2 we look at some of the derived statistics in the GRN. In addition to showing the change in the mean out-degree, the mean in-degree and the number of feed-forward loops, we also show the mean and standard deviation of those statistics for 1000 random network realizations. In the case we call ‘rewired 0.1’, 10% of links are connected to new targets if that new link does not already exist. In the case we call ‘switch 100’, we follow the switch randomization scheme published in (29). In the first case, the number of links remains constant and in the second case, the number of links and the degrees of nodes remain constant. For a detailed discussion of the effects of such mixing algorithms, please refer to our previous work (36). Figure 2 shows that while the number of links increases 1.5 fold, the number of feed-forward loops increases 2-fold. Additionally, we can see that the relative change of the mean number of feed-forward loops in the random networks behaves similarly.

**Figure 3:**
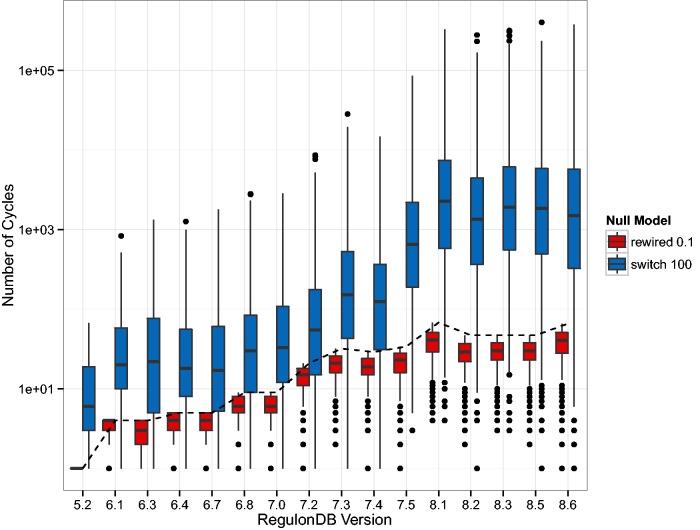
Depicted is the number of elementary circuits of length greater than two, found in the gene–gene regulatory networks (GRN) according to the algorithm in (37). While this number increases drastically (from 1 to 66) in the original networks (dashed line), the increase in the randomized networks is even more dramatic. The boxes in this plot have a horizontal line that represents the median number of elementary circuits and the lower and upper end represent the 25 and 75% quantiles, respectively. The black dots represent outliers. The general trend is that in the switch-randomized networks orders of magnitude more elementary circuits are present, whereas in the partially rewired networks cycles are even more suppressed than in the original GRN.

We also show the number of cycles in the GRN since they may dramatically affect the dynamics of regulation. Due to the large variation in the switch-randomized networks, we follow a different layout, however. In Figure 3, we depicted the absolute number of elementary circuits (37) of length greater than two on a logarithmic scale. The boxplot shows the median number and the 25% and 75% quantiles. These results show that the GRN has a particular topology that when mixed by switch-randomization tends to contain orders of magnitude more cycles. When a fraction of links are rewired, the few cycles that do exist tend to be destroyed.

Having looked at the topological changes, we note the presence of a strong drift in TRN topology. Next, we compare four results from previous studies (24, 26, 30, 32, 34) across the database versions. Three results are purely topology-based and one of them combines gene expression data with the known regulatory architecture.

**Figure 4:**
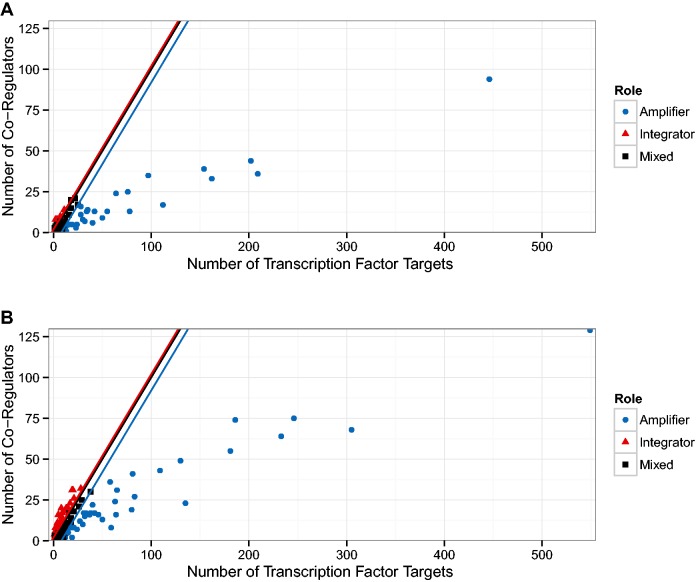
Relationship between the number of targets and co-regulators in the transcriptional regulatory network (TRN). In (24), two populations of transcription factors (TFs) were identified. Those TFs that demonstrate a direct relationship between the number of target genes and the number of other TFs also regulating those genes (co-regulatory partners); and those TFs that have many more target genes than co-regulatory partners. (A) We show a reproduction of Figure 1 g in (24) with the TRN of RegulonDB version 5.2, this is the version available to us that is closest to the TRN used in (24). (B) Shows the figure for the latest version of RegulonDB (8.6). We tracked the fraction of TFs above and below the angle bisector (ignoring a small region around the angle bisector), this is shown in Figure 5. The upper and lower end of that region is depicted by the red and blue line, respectively.

**Figure 5:**
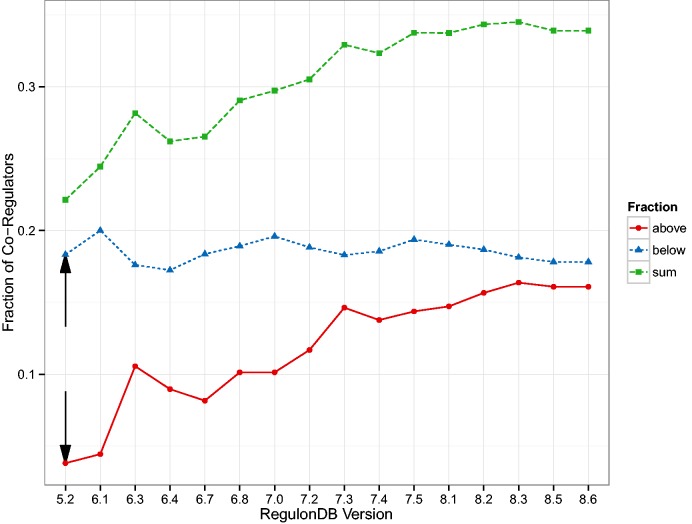
Here, we see the change in the size of the groups identified as in Figure 4 but as a function of the database version. The fraction of TFs that have more co-regulatory partners than target genes (integrators) is increasing. The black arrows depict the values most closely associated with the result in Figure 1 g of (24).

The first result was published in (24) and relates the number of target genes of a TF to the number of co-regulatory partner TFs. In Figure 1 g of (24), the authors identify a group of TFs that observe a 1:1 relationship and another group that have approximately five times more targets than co-regulatory partners. We generalize that result by qualitatively distinguishing between nodes above the diagonal (more co-regulators than targets) and below the diagonal (more targets than co-regulators). We expect a TF above the diagonal to have a functionally different role than those below the diagonal: The ones above are ‘integrators’, involved in several processes and sharing tasks with other regulatory units, while the ones below the diagonal are more decoupled ‘amplifiers’ operating autonomously and affecting a comparatively large number of targets. In Figure 4 we show this separation for the oldest version (5.6) which is similar in size to the one used in (24), as well as for the latest version included in our study (8.6). Both plots look qualitatively similar but Figure 5 reveals a systematic trend towards more integrators, i.e. TFs above the diagonal.

**Figure 6:**
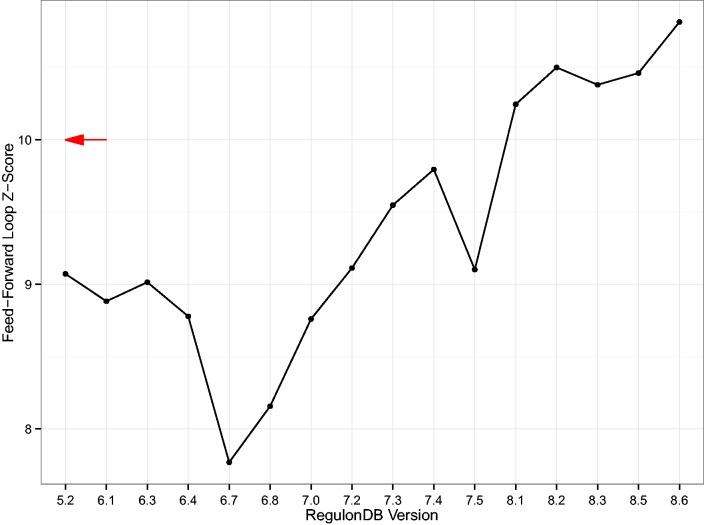
Z-Score of the feed-forward loop. A simple line plot showing the z-score of the three-node feed-forward loop as compared to 1000 switch-randomized gene–gene regulatory networks (GRNs). The z-scores are rather large in magnitude which is a result of the number of feed-forward loops being significantly lower in the random population of networks. Even though the null model, i.e., the switch-randomized networks, are specific for each version, the result still drifts with database version. The red arrow denotes the z-score of the feed-forward loop motif published in (29). That was 4 years prior to the earliest RegulonDB TRN available today.

Second, we computed the over-representation of the feed-forward loop as published in (26, 30) for each database version (Figure 6). We note that the feed-forward loop is significantly over-represented in the GRN in every version and that the large z-scores suggest that the population of random null model networks is topologically far away from the real network. Nevertheless, these results again show a clear drift of results with database version. The result indeed gets more pronounced, providing an additional level of validity.

**Figure 7:**
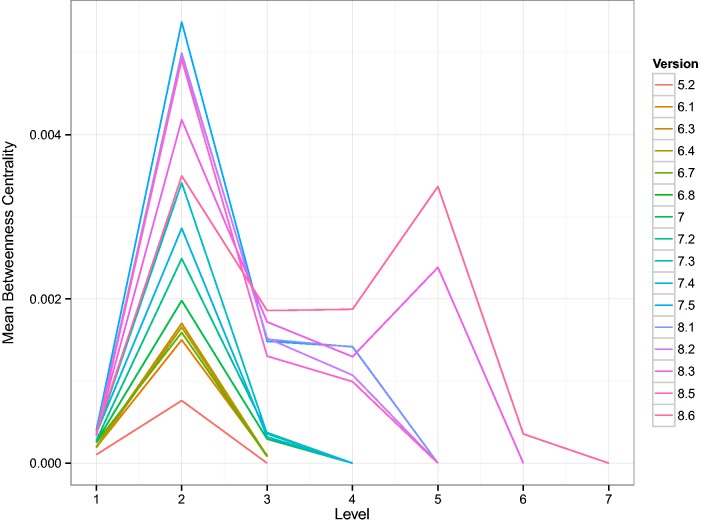
Shown are curves, one for each version of RegulonDB, of the average betweenness centrality of the layers in the TF-hierarchy. Here, 1 is the bottom most layer. The hierarchy was constructed as described in (32). In that study, *E. coli* was reported to have four layers. Here we see an increase in the number of layers as a function of the version and the emergence of a second layer with high average betweenness centrality. This suggests a major discovery of change in the organization of TF–TF regulation.

Following the method published in (32), we induce a hierarchy in the network of TF intra-regulation by defining a bottom layer consisting of TFs that only regulate genes which do not code for other TFs. Additional layers in the hierarchy are then given by the shortest distance of a TF to that bottom layer. Despite using a different TRN as the basis, we reproduce one of their main findings, shown in Figure 7, that the intermediate layers have a high average betweenness centrality. In Ref (32), the authors called those layers bottlenecks of communication or signaling. As might be expected from the growth of the TRN, additional layers appear in the TF hierarchy. It is striking, however, that with the latest two versions a second layer with high average betweenness centrality emerges. That means that in terms of bottlenecks, there are two similar forms of communication layered on top of each other. This result is mirrored by the average out-degree of those TFs in the TRN shown in Supplementary Figure A.2.

Last, we verify the results of a study that investigates functional states of the regulatory system (34). This was done by comparing gene expression data from wild type and mutant *E. coli* with the known architecture of regulatory interactions. As discussed above, the study coined the terms digital control, referring to the binary nature of gene expression caused by TFs, and analog control which describes the continuous range of physical properties of entire chromosomal regions that affect expression. Key results of the original study are: (i) Differences in gene expression due to high (↑σ) and low (↓σ) negative supercoiling, i.e., perturbations of the analog component, reveal the strength of the digital control component. (ii) Disturbing the digital component, achieved by deleting important regulatory hubs of the TRN (FIS and H-NS), exposes considerable analog control strength when comparing the wild type to the mutants. The authors concluded that both modes of control act complementarily.

**Figure 8:**
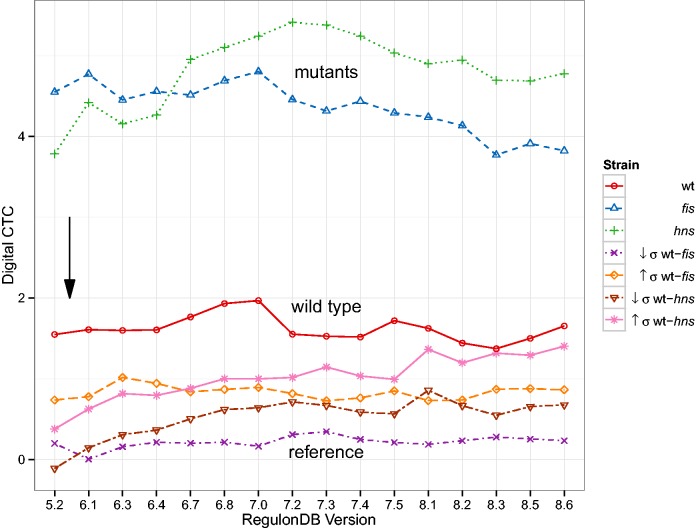
Digital control-type confidence (CTC) as a function of database version. Digital CTC computed as in (34). The black arrow denotes the RegulonDB version (5.6) used in (34). The general separation of results remains remarkably stable. Nonetheless, we can observe some trends in the data: The *hns* mutant strain tends towards higher digital CTC, whereas the *fis* mutant strain decreases in magnitude. The two inter-strain comparisons ↓σ wt-*hns* and ↑σ wt-*hns* also follow an increasing trend.

The digital control type confidence (CTC) shown in Figure 8 is a z-score of digital control measured in a sub network of the TRN selected by differentially expressed genes as compared to $10^{5}$ randomly selected sub networks of equal size. For a detailed description of the methods, please refer to the later Section. The outcomes as a function of the database version behave differently. The general result from the original study (34) remains unchanged. We observe high digital CTC for gene expression data where the analog component was disturbed and this is even more pronounced in the mutants of *fis* and *hns* as compared to the wild type. When contrasting the wild type expression with the mutants, digital CTC is overall insignificant. Looking more closely at the development of these results, however, we see increasing trends for any curves involving *hns* expression data and a decrease in digital CTC for the *fis* curve. These changes are inexplicable purely on the level of the number of regulatory interactions of FIS and H-NS (cf. Supplementary Table A.1).

## Conclusions

We have investigated how the increase in knowledge about regulatory elements (TFs) and interactions contained in RegulonDB over the years from 2006 to 2014 affects exemplary results. The results presented in this study demonstrate that not only reports on topology are immediately affected by content drift, but also results based on content adapted null models. While we have shown that the major conclusions drawn about feed-forward loops in (26, 30) and regulatory control (34) still stand today, we also showed that there are major changes in the organization of TFs. In future work, investigating the change in regulatory organization on the operon level and how it affects the regulation of biological processes seems very promising.

The rise of digital CTC observed in the *hns*-mutant data also illuminates another aspect: the digital and analog control components are entangled more tightly than the results presented suggest. For the sake of completeness, it should be mentioned that H-NS is not only an important global negative regulator in the TRN, but it also is a nucleoid associated protein (NAP) whose role is the silencing of extended chromosomal regions. It therefore provides an important contribution to the digital as well as the analog component. Also, recent chromatin immunoprecipication (ChIP) studies revealed that the extension of the H-NS-silenced chromosomal regions and thus the number of H-NS- repressed genes substantially increases with DNA relaxation on transition to the stationary growth phase (38).

RegulonDB only serves as an example. Similar and possibly stronger effects can be expected from protein-protein interaction networks (PPINs) where experimental techniques and the scope covered in a single experiment changed dramatically over the last decade. In PPINs, the nodes are proteins and a link denotes a physical binding between two proteins. The lack of agreement between different measurements of PPINs has already been widely commented on in the literature, e.g. in (39, 40). Genome-scale metabolic models, which can be seen as a functional database digitally representing an organism’s metabolic system, have also increased substantially in size over the last years (cf. Table 3).

**Table 1.** RegulonDB release history

Version	Release date
5.2	8 June 2006
6.1	15 April 2008
6.3	10 February 2009
6.4	10 August 2009
6.7	24 March 2010
6.8	18 August 2010
7.0	26 January 2011
7.2	6 May 2011
7.3	1 November 2011
7.4	29 March 2012
7.5	29 August 2012
8.1	17 December 2012
8.2	22 April 2013
8.3	29 July 2013
8.5	28 November 2013
8.6	11 April 2014

**Table 2.** Acronyms and abbreviations

Acronym	Meaning
TRN	Transcriptional regulatory network
GPN	Gene proximity network
GRN	Gene–gene regulatory network
GU	Gensor (genetic sensory) unit
TF	Transcription factor
NAP	Nucleoid-associated protein
CTC	Control-type confidence
TU	Transcription unit
sRNA	Small ribonucleic acid

**Table 3.** Summary statistics for a selection of genome-scale metabolic models. Despite a moderate increase in the number of metabolic genes, there is a large increase in the number of modeled reactions and metabolites. For *E. coli*, also see Figure 1 in (41)

Organism	Model	Genes	Reactions	Metabolites
*Escherichia coli*	iJE660a (42)	660	627	438
	iJR904 (43)	904	931	625
	iAF1260 (44)	1260	2077	1039
	iJO1366 (45)	1366	2251	1136
*Saccharomyces cerevisiae*	iFF708 (46)	708	842	584
	iND750 (47)	750	1149	646
	iMM904 (48)	904	1412	1228
	Yeast 5 (49)	918	2110	1655
*Homo sapiens*	Recon 1 (50)	1496	3744	2766
	Recon 2 (51)	1759	7440	5063

## Methods

### Networks

The TRN and GPN were extracted directly from the XML downloads of RegulonDB. The GPN was constructed using gene locations on the chromosome and a window size of 5000 base pairs. Any genes within that window were considered neighbors and were connected with an undirected link in the GPN. The TRN construction was a little more involved. RegulonDB basically includes regulatory interactions between TF conformations and promoters which we reduced to interactions between the underlying TFs and the genes lying in the transcription unit (TU) associated with the promoter. The control analyses were conducted on a directed bipartite network containing potentially multiple links between TFs and genes. For reporting the change in topological measures (see Figure 2) that network was transformed to one containing only genes (the GRN) by replacing the TF nodes with the nodes that represent their coding genes. Table 1 lists the RegulonDB versions analyzed here. In Table 2 frequent abbreviations used throughout this text are listed.

### Microarray data

The expression data are exactly the same as used in (34) and were originally published in (52). They are available at ArrayExpress E-TABM-86 (http://www.ebi.ac.uk/arrayexpress/experiments/E-TABM-86/) but we only used the prepared list of significantly differentially expressed genes. They are gene expression data from wild type *E. coli* LZ41 and LZ54 strains which can be treated to inhibit either DNA gyrase or topoisomerase IV activity and thus affect negative supercoiling (52). Two mutants, *fis* and *hns*, were also studied. A chart describing the organization of the experimental setup can be found in (34) (Figure 1).

### Control analyses

Originally, a control ratio R was computed in an ‘effective’ network, i.e. a sub network generated from only the significantly differentially expressed genes, in either the TRN or GPN. This ratio determines the degree of digital or analog control present in the gene expression data, respectively. R is a ratio between the number of connected nodes $N_{\text{connected}}$ and the number of isolated nodes $N_{\text{isolated}}$,

(1) $$R=\frac{N_{\text{connected}}}{N_{\text{isolated}}}.$$

This ratio is then computed in a population of random ‘effective’ networks that have the same number of nodes as the initial one but where nodes were randomly selected from the complete TRN or GPN. In Ref (34), $10^{4}$ random networks were used to then compute a z-score of R, termed CTC. We have recomputed the results using populations of $10^{5}$ random networks.

(2) $$\text{CTC}=\frac{R-\mu_{R}}{\sigma_{R}},$$

where $\mu_{R}$ and $\sigma_{R}$ are the mean and standard deviation of R in the random sample.

In addition, we have investigated two modifications to computing the digital and analog CTC: (i) The ratio is computed over the total number of nodes $N_{\text{total}}=N_{\text{connected}}+N_{\text{isolated}}$ which makes it a quantity that increases linearly from zero to unity as the number of connected nodes increases,

(3) $$R_{\text{total}}=\frac{N_{\text{connected}}}{N_{\text{total}}}.$$

The original ratio is not defined at $N_{\text{isolated}}=0$ and increases super-linearly as $N_{\text{connected}}\to N_{\text{total}}$. (ii) We adapt the null model for the random ‘effective’ TRNs to maintain the same number of TFs as the original ‘effective’ network. TFs are important hub nodes of high out-degree and a high variability in the number of TFs selected in the random networks dramatically affects the digital control.

The effects of applying these modifications can be seen in the supplementary material. Although we consider them improvements, we excluded these modifications from the main text for the sake of comparability.

The code for parsing the RegulonDB XML data, the expression data and performing the analyses is available as a public git repository (https://github.com/Midnighter/pyorganism). The extensive results are available in HDF5 format from the ‘Computational Systems Biology’ group website (http://sysbio.jacobs-university.de/website/content/data)

### Co-regulation

One of the results in (24) is the relationship between a TF’s number of target genes $N_{\text{targets}}$ and its number of co-regulatory partners $N_{\text{partners}}$, that means, the number of other TFs that also regulate any of the target genes. They then identified two categories of TFs: One where $N_{\text{partners}}\propto N_{\text{targets}}$ and another one where $5N_{\text{partners}}\propto N_{\text{targets}}$. For each RegulonDB release, we computed the fraction of TFs where $N_{\text{partners}}>N_{\text{targets}}+2$, i.e., above a minimal distance from the angle bisector, and the fraction of TFs where $N_{\text{partners}}<N_{\text{targets}}-8$, i.e. below a proportional distance.

## Funding

M.E.B. was supported by a grant from Deutsche Forschungsgemeinschaft (DFG) to MTH (grant no. HU-937/6). MTH also acknowledges support from BMBF (e:med program, grant 01ZX1306D). Funding for open access charge: BMBF (e:med program, grant 01ZX1306D).

## Supplementary data

Supplementary data are available at *Database* Online. 

*Conflict of interest*. None declared.

Supplementary Data
