# Supplementary material for: Effect of database drift on network topology and enrichment analyses: a case study for RegulonDB
Source: Database (Oxford). 2016 Mar 15;2016:baw003. doi: 10.1093/database/baw003 (PMC4792529; doi:10.1093/database/baw003)
Supplement: Supplementary Data [file supp_2016_baw003_index.html]

Effect of database drift on network topology and enrichment analyses: a case study for RegulonDB — Supplementary Data 

# Effect of database drift on network topology and enrichment analyses: a case study for RegulonDB

## Supplementary Data

files

- Supplementary Data - pdf file
