# Supplementary material for: Effect of database drift on network topology and enrichment analyses: a case study for RegulonDB
Source: Database (Oxford). 2016 Mar 15;2016:baw003. doi: 10.1093/database/baw003 (PMC4792529; doi:10.1093/database/baw003)
Supplement: Supplementary Data [file supp_baw003_supplements.pdf]

Complete Control Statistics per RegulonDB Version

Moritz E. Beber[†‡], Georgi Muskhelishvili[†] and Marc-Thorsten Hütt[†]

[†]Department of Life Sciences and Chemistry, Jacobs University, Campus Ring 1, 28759 Bremen, Germany
[‡]Bioinformatics Group, Max Planck Institute for Molecular Genetics, Ihnestraße 63–73, 14195 Berlin, Germany

Network statistics

While the main text shows only a few notable changes in the TRN (see Figure 2), Table A.1 & A.2 contain the full information that we investigated and especially information on the GPN. In addition to the information displayed before, Table A.1 contains the density of the TRN, the out-degree of both FIS and H-NS, the maximum out-degree, the degree associativity (out-in), the number of components and the size of the largest connected component (LCC). Most topological statistics in the TRN follow an increasing trend. Exceptions to this rule are the density, which means that proportionally more nodes have been added than links, the mean in- and out-degree and the degree associativity.

The topology of the GPN remains fairly constant over the observed range. The only exception is the number of components in version 7.0. This can also be seen in Figure A.6 where this fact manifests as a tiny peak but seems uninteresting otherwise.

Table A.1: Topological characteristics of the TRN and GRN in the successive RegulonDB versions. Unless mentioned otherwise the numbers refer to the GRN. Showing from left to right: the number of genes defined in RegulonDB (Genes), the number of transcription factors defined in RegulonDB (TFs), the number of nodes (Nodes), the number of links (Links), the network density (Density), the mean out-degree of all nodes with out-degree larger than zero ($\langle k_{\text{out}} \rangle$), the mean in-degree of all other nodes ($\langle k_{\text{in}} \rangle$), the out-degree of FIS in the TRN (FIS), the out-degree of H-NS in the TRN (H-NS), the maximum out-degree ($\max(k_{\text{out}})$), the degree associativity between out- and in-degree (Ass. (out-in)), the number of weakly connected components (Comp.), the largest of those connected components (LCC), the number of auto-regulatory loops (Auto), the number of feed-forward loops (FFLs), the number of elementary circuits in the network (Cycles), the release date (Release) and the respective version (Version).

Genes	TFs	Nodes	Links	Density	$\langle k_{\text{out}} \rangle$	$\langle k_{\text{in}} \rangle$	FIS	H-NS	$\max(k_{\text{out}})$	Ass. (out-in)	Comp.	LCC	Auto	FFLs	Cycles	Release	Version
4517	150	1307	2898	0.0017	18.9	2.38	162	97	396	0.0697	18	1203	92	812	1	2006-06-08	5.2
4579	160	1476	3394	0.001 56	20.8	2.45	172	141	413	0.0602	23	1376	104	843	4	2008-04-15	6.1
4579	166	1523	3525	0.001 52	20.9	2.46	220	148	421	0.0547	22	1432	109	902	4	2009-02-10	6.3
4602	169	1546	3580	0.0015	20.6	2.47	220	149	424	0.0541	23	1452	113	905	5	2009-08-10	6.4
4605	176	1599	3696	0.001 45	20.9	2.45	224	150	436	0.0501	23	1506	118	959	5	2010-03-24	6.7
4605	175	1627	3772	0.001 43	21.1	2.46	224	157	436	0.0362	23	1532	120	1015	9	2010-08-18	6.8
4621	177	1633	3841	0.001 44	21.3	2.49	224	157	437	0.031	24	1536	122	1062	9	2011-01-26	7.0
4622	179	1635	3921	0.001 47	21.5	2.54	222	170	437	0.0245	21	1551	125	1140	21	2011-05-06	7.2
4635	188	1686	4000	0.001 41	21.6	2.5	227	173	459	0.01	22	1596	127	1337	32	2011-11-01	7.3
4635	190	1700	4071	0.001 41	21.7	2.52	227	174	473	0.0137	21	1613	130	1430	29	2012-03-29	7.4
4634	185	1697	4100	0.001 42	22.4	2.54	227	174	477	0.001 94	20	1615	128	1437	34	2012-08-29	7.5
4637	188	1717	4178	0.001 42	22.5	2.55	227	180	480	−0.0258	22	1628	129	1556	68	2012-12-17	8.1
4637	201	1735	4167	0.001 39	22	2.53	227	179	481	−0.0141	23	1642	130	1489	47	2013-04-22	8.2
4639	202	1746	4199	0.001 38	22.1	2.53	229	179	481	−0.0157	23	1653	131	1506	47	2013-07-29	8.3
4640	202	1769	4217	0.001 35	22.2	2.5	229	179	494	−0.0171	23	1676	131	1494	47	2013-11-28	8.5
4639	202	1791	4310	0.001 34	21.8	2.52	233	186	496	−0.0228	24	1694	138	1578	66	2014-04-11	8.6

Table A.2: Topological characteristics of the GPN in the successive RegulonDB versions. Showing from left to right: the number of genes defined in RegulonDB (Genes), the number of nodes (Nodes), the number of undirected links (Links), the network density (Density), the mean degree ($\langle k \rangle$), the maximum degree ($\max(k)$), the degree associativity (Ass.), the number of connected components (Comp.), the largest of those connected components (LCC), the release date (Release) and the respective version (Version).

Genes	Nodes	Links	Density	$\langle k \rangle$	$\max(k)$	Ass.	Comp.	LCC	Release	Version
4517	4517	25048	0.002 46	11.1	24	0.741	12	4506	2006-06-08	5.2
4579	4579	25861	0.002 47	11.3	24	0.748	6	4574	2008-04-15	6.1
4579	4579	25875	0.002 47	11.3	24	0.747	5	4575	2009-02-10	6.3
4602	4602	26149	0.002 47	11.4	24	0.745	5	4598	2009-08-10	6.4
4605	4605	26190	0.002 47	11.4	24	0.745	5	4601	2010-03-24	6.7
4605	4605	26202	0.002 47	11.4	24	0.745	4	4602	2010-08-18	6.8
4621	4621	26202	0.002 45	11.3	24	0.745	20	4602	2011-01-26	7.0
4622	4622	26386	0.002 47	11.4	24	0.744	4	4619	2011-05-06	7.2
4635	4635	26517	0.002 47	11.4	24	0.742	4	4632	2011-11-01	7.3
4635	4635	26521	0.002 47	11.4	24	0.742	4	4632	2012-03-29	7.4
4634	4634	26511	0.002 47	11.4	24	0.742	4	4631	2012-08-29	7.5
4637	4637	26547	0.002 47	11.5	24	0.741	4	4634	2012-12-17	8.1
4637	4637	26547	0.002 47	11.5	24	0.741	4	4634	2013-04-22	8.2
4639	4639	26568	0.002 47	11.5	24	0.74	4	4636	2013-07-29	8.3
4640	4640	26578	0.002 47	11.5	24	0.741	4	4637	2013-11-28	8.5
4639	4639	26568	0.002 47	11.5	24	0.74	4	4636	2014-04-11	8.6

Motif Signatures

Using the mfinder software (version 1.21 `http://www.weizmann.ac.il/mcb/UriAlon/download/network-motif-software`) [2], over all version of the TRN six different three-node subgraph motifs are found: the feed-forward loop (ID 38), its two related subgraphs where one of the links is bidirectional (ID 46 and 108), the two subgraphs that, due to their bidirectional links, could function either as a feed-forward or feedback loop (ID 102 and 110) and the clique (ID 238). The last three subgraphs occur very rarely (0–3 unique occurrences) and the true feedback loop (ID 98) does not occur in any of the RegulonDB versions.

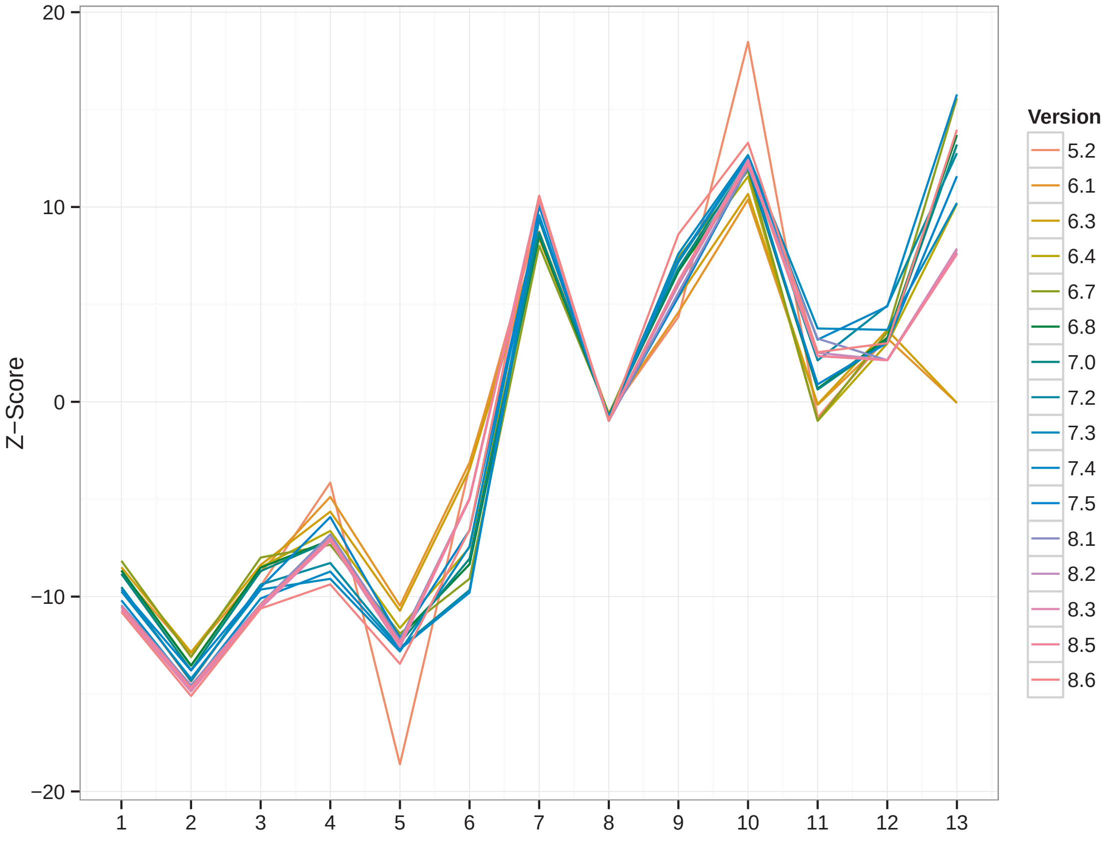

Figure A.1: Despite the large magnitude of Z-scores, mfinder [2] identifies only six motifs: the feed-forward loop (ID 38, position 7), the two structurally similar subgraphs (ID 46 and 108, at positions 10 and 9), subgraphs with ID 102 and 110 (positions 11 and 12) and the clique (ID 238, position 13). The feedback loop (ID 98, position 8) does not occur in any version. The ordering of the subgraphs corresponds to [1].

Transcription Factor Hierarchy

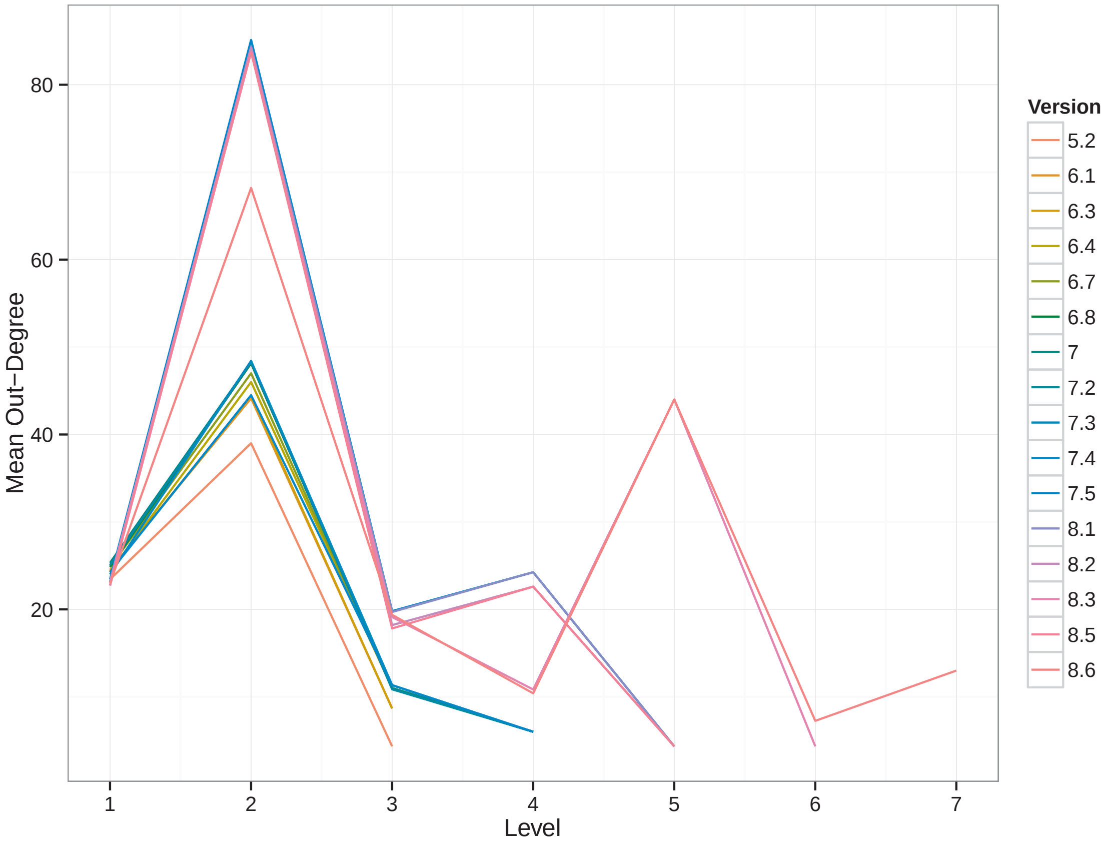

Figure A.2: Shown are curves, one for each version of RegulonDB, of the average out-degree of TFs in the TRN as a function of the layers they occupy in the TF-hierarchy. Here, 1 is the bottom most layer. The hierarchy was constructed as described in [3]. In that study, *E. coli* was reported to have four layers. Here we see an increase in the number of layers as a function of the version and the emergence of a second layer with high average out-degree. This suggests a major discovery of change in the organization of TF-TF regulation by additional regulatory data.

Control strength

Overview of the raw digital and analog control strength as a function of the RegulonDB version. Both ratios discussed (1) and (3) are shown in Figures A.3 & A.4.

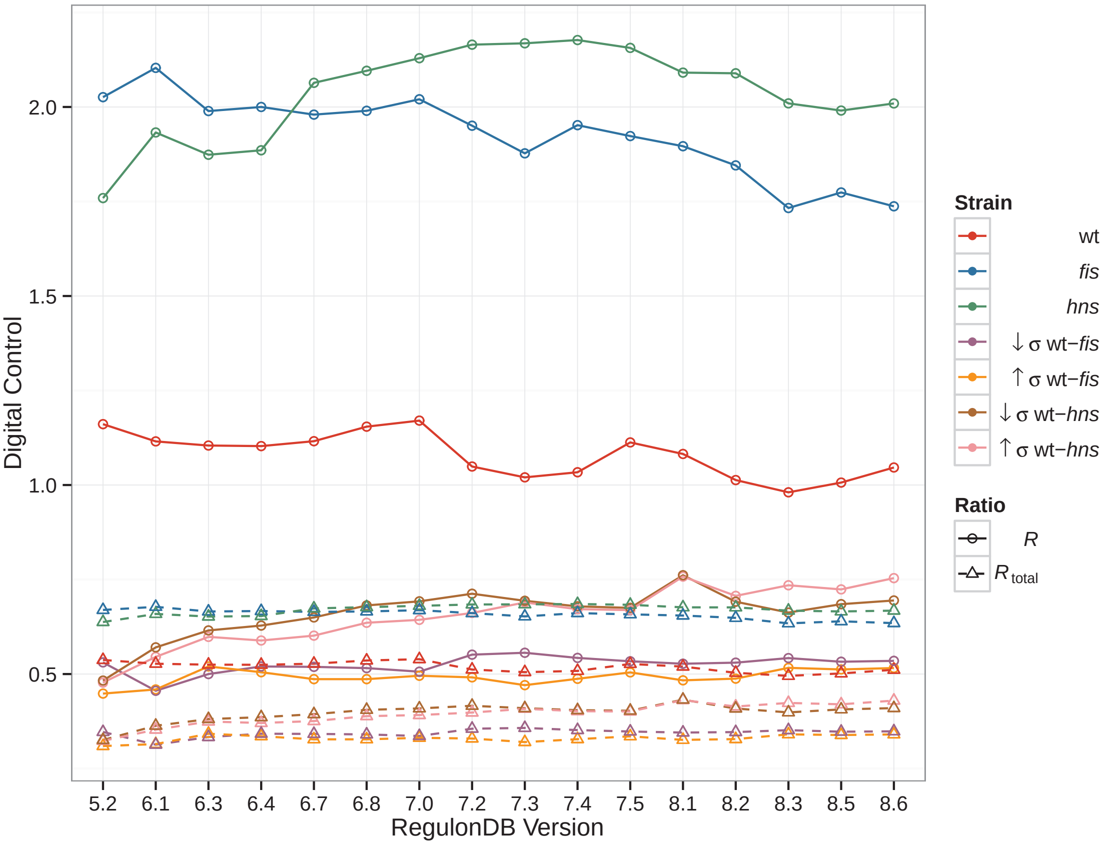

Figure A.3: Digital control strength of the 'effective' TRNs computed with the original R (1) and the new ratio R_{total} (3). We can clearly see that all profiles are shifted between zero and unity for R_{total} but that their relative order is unchanged. The figure shows that the maximum ratio of connected nodes is $\approx \frac{2}{3}$ and the minimum $\approx \frac{1}{3}$.

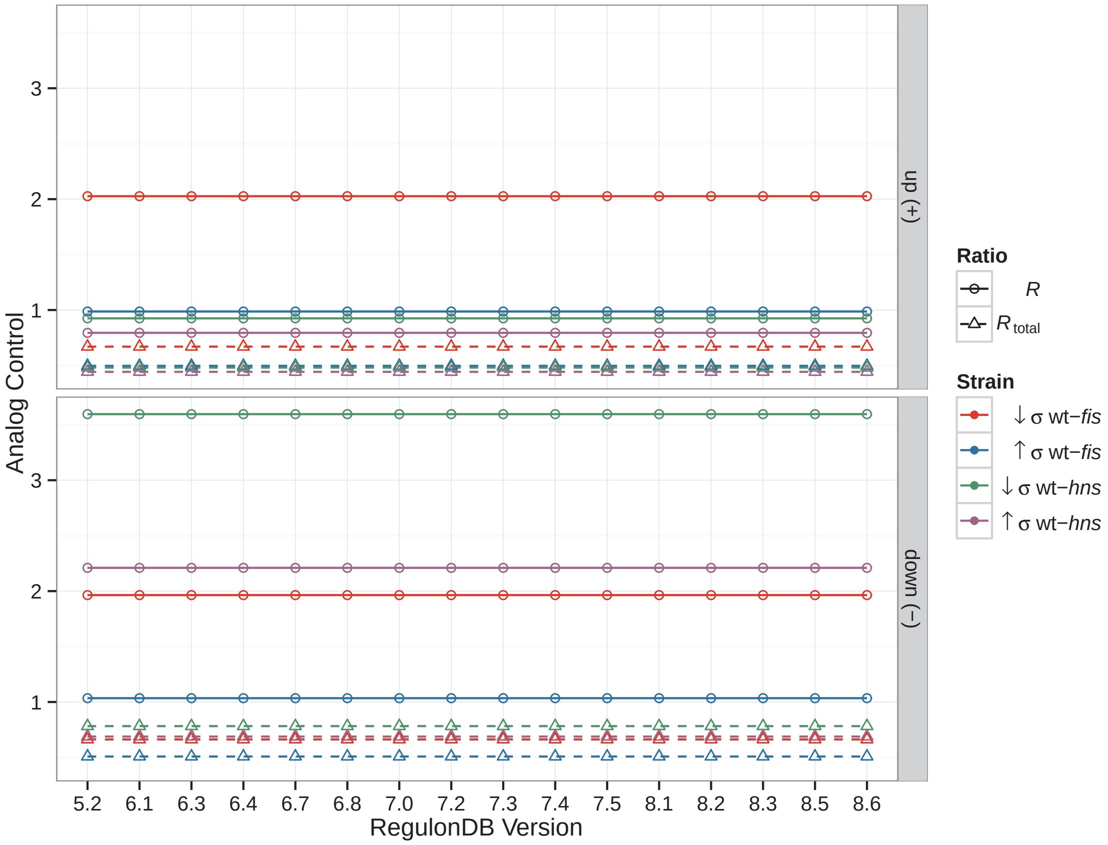

Figure A.4: Analog control strength of the 'effective' GPNs computed with the original R (1) and the new ratio R_{total} (3) depicted separately for the positive (+) and negative (−) $\log_2$ expression ratios. We can clearly see that all profiles are shifted between zero and unity for R_{total} but that their relative order is unchanged. The figure shows that the maximum ratio of connected nodes is ≈ 0.8 and the minimum $\approx \frac{1}{2}$.

Control type confidence

Overview of digital, analog and ΔCTC as a function of the RegulonDB version. Both ratios discussed (1) and (3) are shown in Figures A.5, A.6 & A.7. Figure A.5 also includes the original and improved null model discussed in Section .

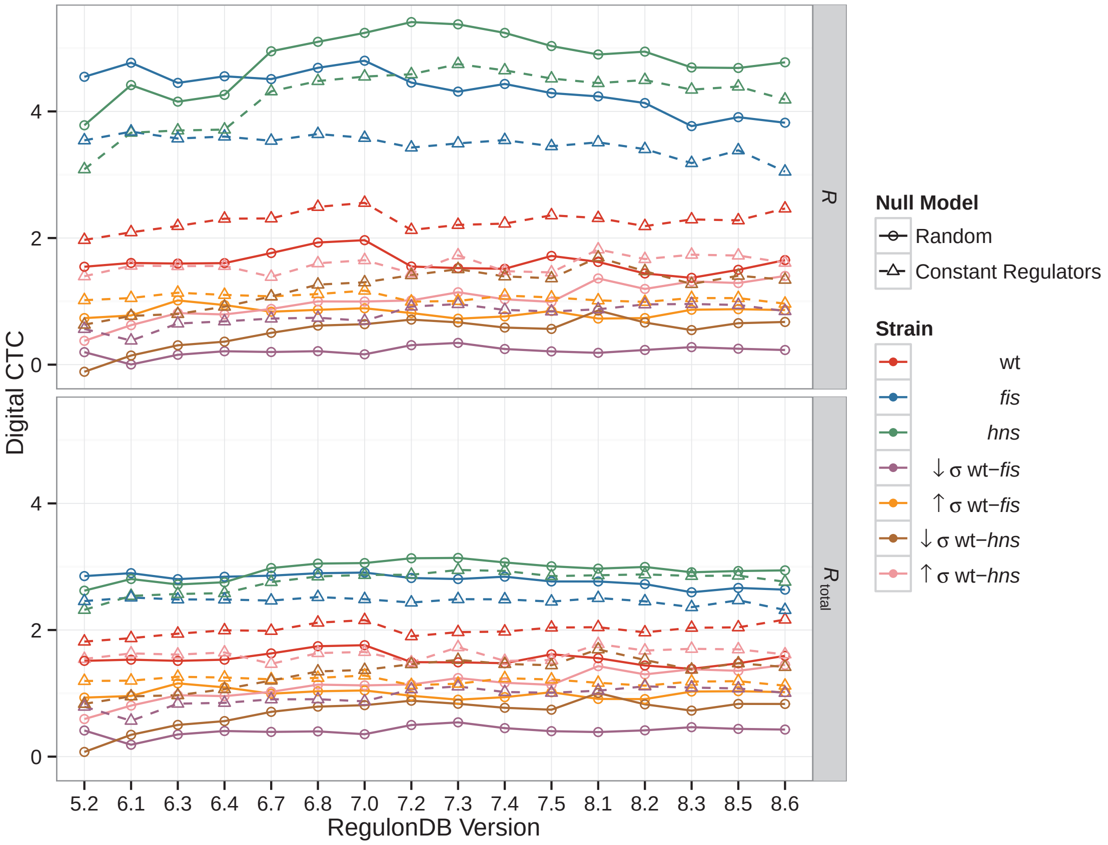

Figure A.5: Digital control type confidence of the two null models discussed in Section , shown here for both ratios: (top) R (1) and (bottom) R_{total} (3). These results clearly show that both changes to the computation of the CTC have a mostly dampening effect. The dashed lines in the lower panel correspond to Figure 8.

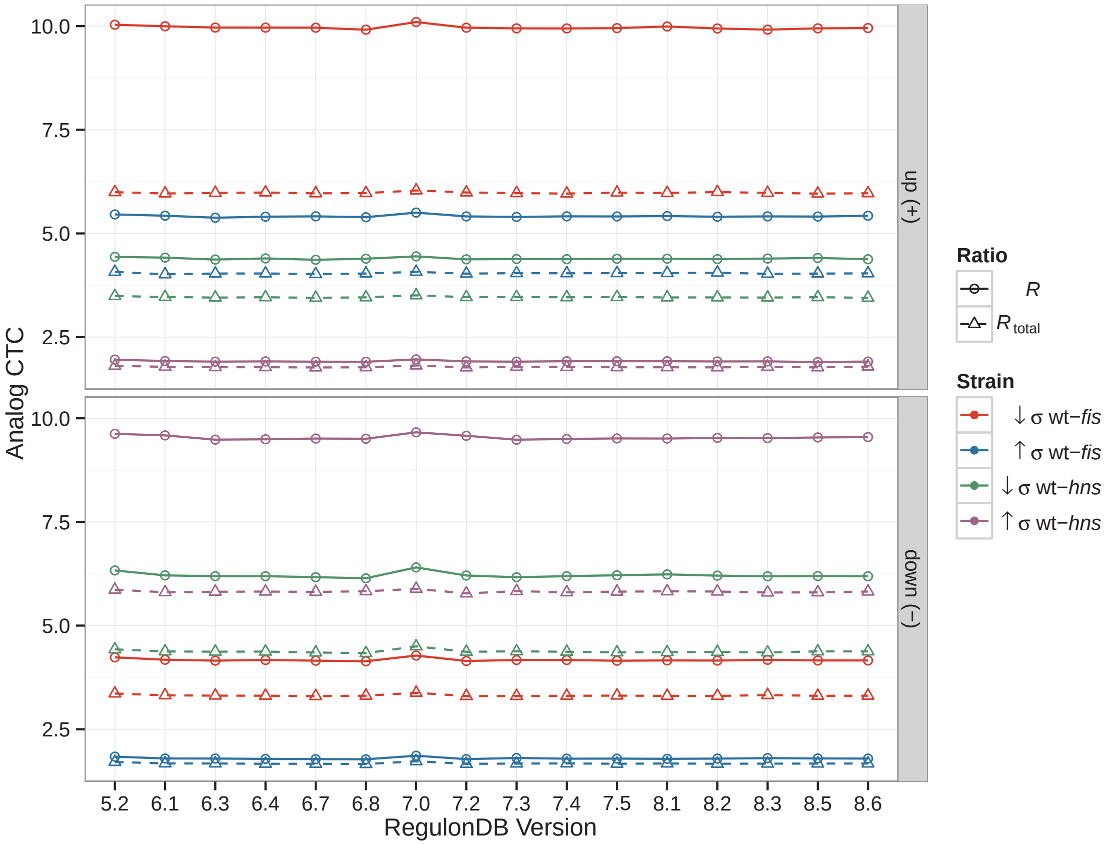

Figure A.6: Analog control type confidence of both ratios: R (1) and R_{total} (3), shown separately here for positive (+) and negative (−) $\log_2$ expression ratios. These results clearly show the overall reduced magnitude in CTC caused by employing the updated ratio. The tiny peak at version 7.0 is probably related to GPN structure that is unique to that version (also cf. Table A.2).

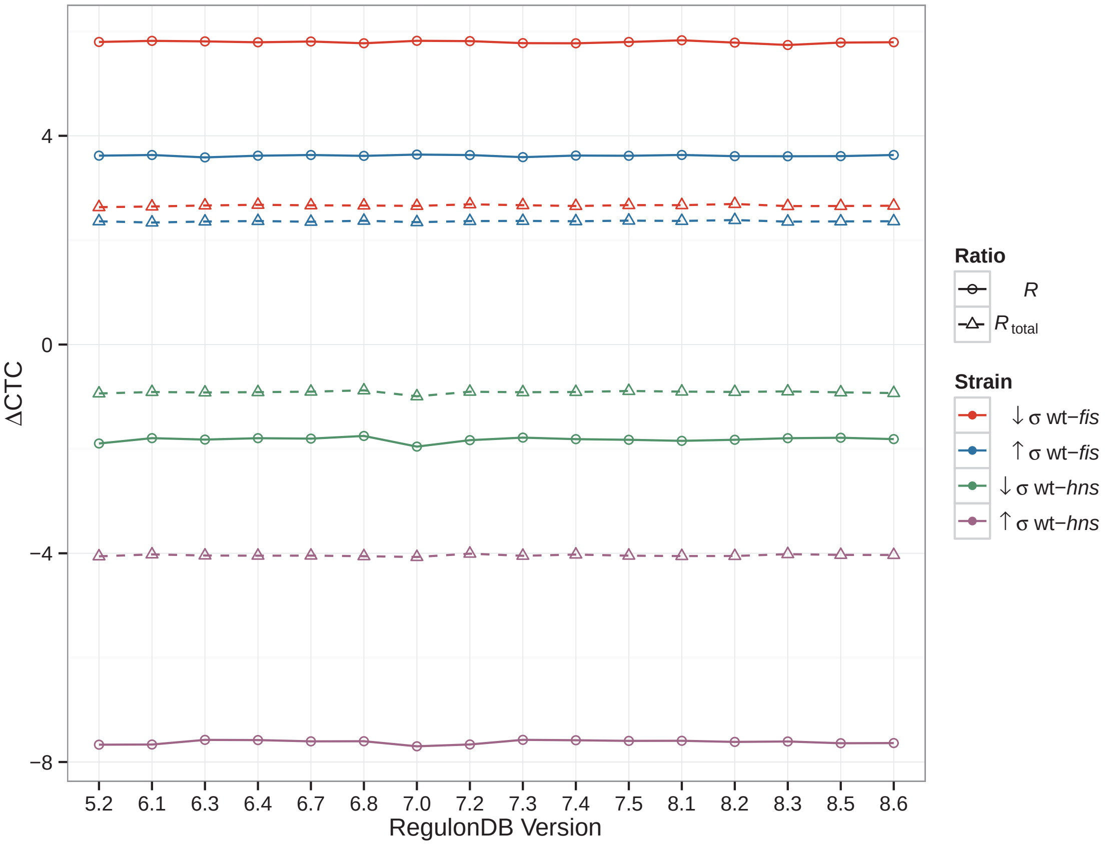

Figure A.7: Δ control type confidence of both ratios: R (1) and R_{total} (3). They are the difference of the respective analog CTC (+) and (−). These results clearly show the overall reduced absolute value in ΔCTC caused by employing the updated ratio.

References

[1] R. Milo, S. Itzkovitz, N. Kashtan, R. Levitt, S. Shen-Orr, I. Ayzenshtat, M. Sheffer, and U. Alon. Superfamilies of evolved and designed networks. *Science*, 303(5663):1538, 2004.

[2] R. Milo, S. Shen-Orr, S. Itzkovitz, N. Kashtan, D. Chklovskii, and U. Alon. Network motifs: Simple building blocks of complex networks. *Science*, 298(5594):824–827, October 2002.

[3] Haiyuan Yu and Mark Gerstein. Genomic analysis of the hierarchical structure of regulatory networks. *Proceedings of the National Academy of Sciences of the United States of America*, 103(40):14724–14731, October 2006.
